# Proteomic Insights into Cardiac Fibrosis: From Pathophysiological Mechanisms to Therapeutic Opportunities

**DOI:** 10.3390/molecules27248784

**Published:** 2022-12-11

**Authors:** Ruiqiang Qi, E. Lin, Juan Song, Yan Wang, Ling Lin

**Affiliations:** 1 Xiamen Cardiovascular Hospital of Xiamen University, School of Medicine, Xiamen University, Xiamen 361000, China; 2 School of Cancer and Pharmaceutical Studies, King’s College London, London SE19RT, UK; 3 Shanghai Institute of Cardiovascular Diseases, Zhongshan Hospital, Fudan University, Shanghai 200032, China

**Keywords:** cardiac fibrosis, fibroblast activation, extracellular matrix, circulating biomarkers, therapeutic strategies, cell-type resolved proteomics

## Abstract

Cardiac fibrosis is a common pathophysiologic process in nearly all forms of heart disease which refers to excessive deposition of extracellular matrix proteins by cardiac fibroblasts. Activated fibroblasts are the central cellular effectors in cardiac fibrosis, and fibrotic remodelling can cause several cardiac dysfunctions either by reducing the ejection fraction due to a stiffened myocardial matrix, or by impairing electric conductance. Recently, there is a rising focus on the proteomic studies of cardiac fibrosis for pathogenesis elucidation and potential biomarker mining. This paper summarizes the current knowledge of molecular mechanisms underlying cardiac fibrosis, discusses the potential of imaging and circulating biomarkers available to recognize different phenotypes of this lesion, reviews the currently available and potential future therapies that allow individualized management in reversing progressive fibrosis, as well as the recent progress on proteomic studies of cardiac fibrosis. Proteomic approaches using clinical specimens and animal models can provide the ability to track pathological changes and new insights into the mechanisms underlining cardiac fibrosis. Furthermore, spatial and cell-type resolved quantitative proteomic analysis may also serve as a minimally invasive method for diagnosing cardiac fibrosis and allowing for the initiation of prophylactic treatment.

## 1. Introduction

Cardiovascular diseases (CVDs) remain the predominant cause of global mortality for all males and females, and the burden of CVD continues the decades-long rise for almost all countries [1]. Cardiac fibrosis, characterized by excessive fibrillar collagen synthesis and deposition, is a common pathophysiologic process in most CVDs (e.g., different types of cardiomyopathies, hypertensive heart disease, diabetic heart disease, and myocardial infarction) [2]. Under multiple injuries and stimuli, including myocardial hypoxia, pressure overload, pathogen infection, metabolic dysfunction and aging, the over-activation of fibroblasts present a critical step in the development of cardiac fibrosis and lead to myocardial remodeling as well as interstitial collagen deposition [3,4]. With the increasing of abnormal collagen around cardiomyocytes, myocardial fibrosis leads to left ventricular diastolic dysfunction, arrhythmia, and impaired myocardial oxygen availability, eventually triggers end-stage heart failure even with adequate clinical management [5,6]. Thus, early in heart injury, reactive fibrosis and collagen remodeling occur in the absence of necrosis, whereas reparative fibrosis presents later on. Despite enormous advances in deciphering the molecular mechanisms underlying cardiac fibrosis, the cell-type specific adaptations and intercellular signaling crosstalk remain poorly understood. In this review, we summarize the current understandings of cardiac fibrosis, demonstrate novel diagnostic biomarkers of advanced fibrotic heart disease, and explore the potential therapeutic targets for earlier intervention to restore healthy remodeling.

## 2. Fundamentals of Cardiac Fibrosis

### 2.1. The Histopathological Types of Cardiac Fibrosis

**Figure 1:**
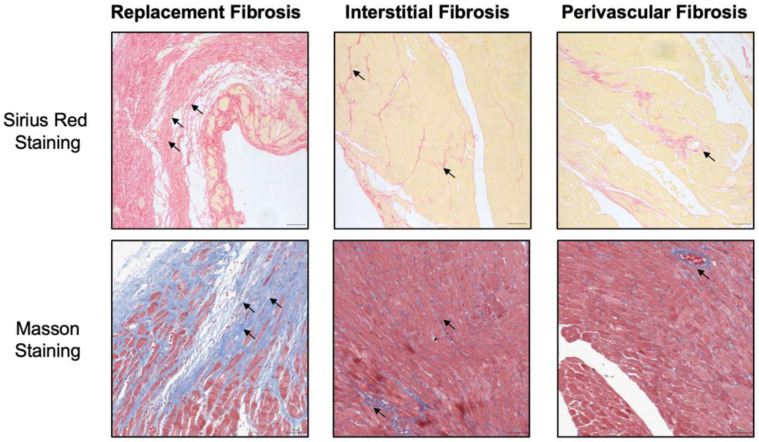
Three histopathological types of cardiac fibrosis via sirius red staining and masson staining. Black arrow showed increased collagen accumulation in cardiac tissue. Sirius red staining visualized collagen fibers selectively, while masson staining exhibited fibrosis blue and muscle red.

Cardiac fibrosis is characterized by excessive cross-linking and deposition of collagens and other extracellular matrix (ECM) proteins, and the pathological types include replacement fibrosis, interstitial fibrosis and perivascular fibrosis (Figure 1) [7,8]. Myocardial infarction (MI) is a typical example of replacement fibrosis, as sudden death of a huge number of cardiomyocytes triggers inflammation and subsequently activates fibroblasts, leading to formation of a collagen-based scar. Protective scar at the infarct area maintains the structural integrity of the chamber, and prevents cardiac rupture—a fatal consequence of MI. However, the infarct scar lacks contractile capacity, and cardiomyocytes within the non-infarcted areas suffer disturbed homeostasis and intrinsic signaling defects because of the extensive collagen secretion which is detrimental to cardiac function [9,10]. As for interstitial fibrosis, numerous ongoing long-run maladaptive stimulations, such as pressure or volume overload [11,12], aging [13], metabolic disorders [14,15] and toxic substances [16], usually lead to interstitial fibrosis and perivascular fibrosis in the absence of cardiomyocyte death. Pressure overload diseases including primary or secondary hypertension and aortic stenosis are predominant cause of interstitial myocardial fibrosis. Research group of Robert Blanton revealed that adult male mice exhibited different degree of systolic dysfunction and cardiac fibrosis, which were randomly received transverse aortic constriction (TAC) surgery with varying degrees of tightness [17]. Specifically, TAC severity resulted in a graded response of cardiac hypertrophy, dysfunction and fibrosis. The excessive deposition of collagens (e.g., collagen I and collagen III) reduces myocardial elasticity, causing disorders in the systolic and diastolic capacity of the heart, ultimately leading to the heart failure [6]. In addition, cardiomyocytes construct an electrical syncytium in healthy heart. Collagen fibers produced by the wound-healing process has the detrimental consequences of disrupting the electrical coupling between adjacent strands of myocytes which contribute to the development of nausea arrhythmias [18]. All these distinct types of fibrosis could be present in parallel within the same heart, making the chances for sampling error in clinic. As many fibrotic features are shared and feasibly transitioned into one another, anti-fibrotic therapy should be personalized according to the diagnosis of specific and probably dominant type of fibrosis.

### 2.2. Imaging and Biochemical Methods to Detect Cardiac Fibrosis

Endomyocardial biopsy is the diagnostic gold standard for cardiac fibrosis, which mainly detects and quantifies the collagen of interstitial [19,20,21]. However, biopsy only reflects the interstitial myocardium at the site of sampling and is an invasive test for patients which hinders the wide-scale clinical application [22]. Non-invasive diagnosis of myocardial fibrosis is also used in clinical practice. Imaging methods, including echocardiography, cardiac magnetic resonance and nuclear imaging are routinely used to evaluate the presence, scope, and turnover of cardiac fibrosis [23,24]. By measuring changes of myocardial structure and function, valvules, and hemodynamics, echocardiographic imaging provides an overview of the heart situation. The accuracy of imaging-based diagnosis relies on the quality of the images and the doctors’ experience [25]. Cardiac magnetic resonance is another popular diagnostic imaging method. Late-enhancement magnetic resonance imaging (MRI) currently provides the best depiction of fibrotic lesions. By applying the gadolinium-based contrast agents, MRI enables cardiologists to distinguish the components of the interstitial myocardium and assess cardiac scar fibrosis [26,27]. In addition to imaging approaches, a number of circulating biomarkers have been proposed for the non-invasive assessment of cardiac function and myocardial fibrosis [22,28].

Excessive myocardial collagen cross-linking determines myocardial collagen’s resistance to degradation by matrix metalloproteinase-1 (MMP-1) and interstitial accumulation of collagen fibers with impairment of cardiac function. Querejeta et al. investigated whether increased collagen type I synthesis and deposition contribute to deterioration of cardiac function, and proved increased circulating levels of serum carboxy-terminal propeptide of procollagen type I (PICP) could be a promising marker for diagnosis of severe myocardial fibrosis [29]. Combination of PICP blood levels and late gadolinium enhancement at cardiac magnetic resonance provided additional prognostic information of myocardial fibrosis in dilated cardiomyopathy patients [30]. Park et al. discovered a significant reduction of full-length carboxy-terminal telopeptide of collagen type I (CITP) in serum levels from patients with heart failure, and suggested this protein as a potential circulating biomarker for assessment of cardiac fibrosis [31]. Research group of Réka Faludi currently addressed that galectin-3, a carbohydrate binding lectin secreted by activated macrophages, potentially provided information reflecting the integrated effects of fibrosis, inflammation, and left ventricular systolic function [32]. Notably, none of the circulating biomarkers are implemented in clinical practice to date. To develop strategies aimed to validate biomarkers of myocardial fibrosis useful in the clinical handling of cardiac patients, collaborations between clinical medicine, biochemical testing and omics analysis are needed. The combination of imaging and circulating biomarkers may integrate different levels of information, overcome methodological limitations, and contribute to a better profiling of each individual patient with a view to personalize the therapy.

### 2.3. Emerging Therapeutic Strategies for Cardiac Fibrosis

Nearly 45% of all deaths in the developed world are attributed to some type of chronic fibroproliferative disease [33]. It is important to reverse progressive fibrosis and restore normal tissue architecture when developing novel antifibrotic strategies. In the initial stage of fibrosis formation, protection of the myocardium from fibrosis, such as antihypertension, valve replacement, and coronary intervention, are the best ways to limit the progression of fibrosis without adversely affecting the overall repair process. Currently, clinical practice focuses on inhibiting myocardial fibrosis by pharmacological blocking the activation of renin-angiotensin-aldosterone system (RAAS) [34]. Various blockers targeting the RAAS, such as renin inhibitors, ACE inhibitors, angiotensin receptor blockers and aldosterone antagonists, represent effective in preventing fibroblast activation and cardiac remodeling. Transforming growth factor-β (TGF-β), the principal mediator of fibrosis, is also an attractive therapeutic target for drug development. By interrupting the TGF-β ligand, the interaction of ligand–receptor and intracellular signaling, candidate molecules disturb the downstream signaling of TGF-β-related proteins [35]. Although there is a multitude of evidence showing the antifibrotic effects of TGF-β inhibition, the clinical application has been limited because of the paradoxical influence of TGF-β inhibition [33,36]. Given TGF-β signaling serves as an energizer or a suppressor in multiple disorders, organ-specific molecular targets need to be developed. In addition to medication, biomaterials have been clinically applied to antifibrosis treatment. Yao et al. have reported a cardiac patch integrating reactive oxygen species (ROS)-scavenging which reduced MI-induced cell apoptosis, suppressed inflammatory response, and alleviated fibrosis [37].

## 3. Cell Biological Mechanisms Underlining Cardiac Fibrosis

### 3.1. The Cellular Effectors of Cardiac Fibrosis

**Figure 2:**
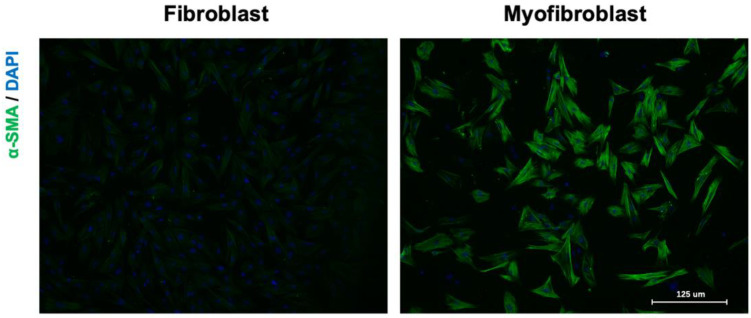
Fibroblast-to-myofibroblast transition. Primary cells were stained with anti-αSMA antibody (green), and cell nuclei were stained with 4′,6-diamidino-2-phenylindole (blue).

Human heart is composed of five major cell types: cardiomyocytes, fibroblasts, endothelial cells, macrophages, and smooth muscle cells [38,39]. Cardiomyocytes contribute roughly 30% to 40% by number and roughly 65% to 80% by volume in the adult mammalian heart [40]. Cardiac fibroblasts physiologically maintain the dynamic balance of ECM synthesis and degradation and account for only 25% of normal myocardial tissue volume, but they are one of the most abundant cell types present in human heart; they occupy approximately 60–70% of cell numbers [41]. They have been termed as sentinel cells because fibroblasts can sense extracellular environment changes, such as chemical, mechanical, and electrical signals in the heart, and make the appropriate response [42]. The activated fibroblasts and myofibroblasts are the principal cellular effectors in cardiac fibrosis, which serve as the primary source of matrix proteins [3]. Concretely, cardiac fibroblasts are activated into myofibroblasts with an increasing proliferative and invasive capacity upon cardiac injury (Figure 2). This conversion of fibroblast is termed as fibroblast-to-myofibroblast transition (FMT), making myofibroblasts produce ECM proteins, including collagens, glycoproteins and proteoglycans (e.g., fibronectin, periostin, and galectins), to initially provide local mechanic support to the failing heart [43]. Activated myofibroblasts can also contribute to the regulation of ECM remodeling by producing proteases, such as matrix metalloproteinases (MMPs), and their inhibitors. Excessive accumulation of ECM components leads to environmental stiffness and further affects structural and functional properties of the myocardium. Increased mechanical forces can lead to cardiomyocyte damage and pathological fibrosis [44]. In addition to fibroblasts and cardiomyocytes, endothelial cells [45], epicardial cells [46] and immunocytes [47] also participate in the occurrence and progression of myocardial fibrosis. Together, fibroblasts play a critical role in cardiac fibrosis despite the fact that there are different phenotypic forms induced by various etiologies.

### 3.2. The Master Regulators of Cardiac Fibrosis

Cardiac fibrosis and FMT process are regulated by multiple cytokines and inflammatory factors. The RAAS is activated during cardiac fibrosis and communicates with signaling pathways contributing to fibrosis [48]. Angiotensin II (Ang II) mediates phenotype switching of cardiac fibroblasts into myofibroblasts through binding to Ang II receptor type 1 (AT1R) [49]. Ang II also stimulates fibroblast to release exosomes, which in turn increases Ang II production and elevates its receptor expression in cardiomyocytes [50]. Positive feedback between exosomes and Ang II exacerbates myocardial remodeling.

**Figure 3:**
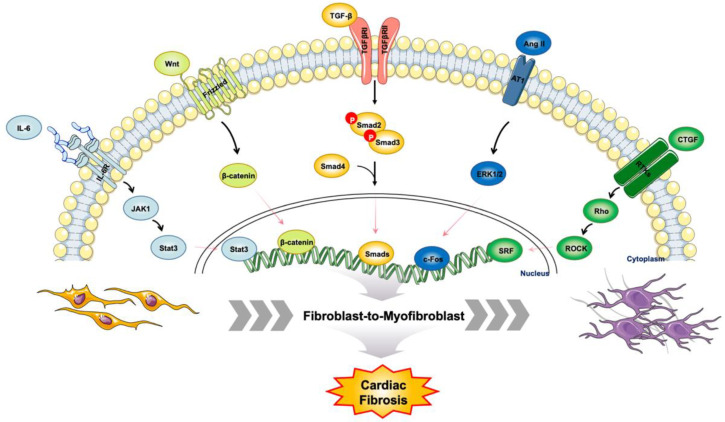
Cellular signaling crosstalk involved in the regulation of cardiac fibrosis.

TGF-β1, a potent fibrogenic cytokine which initiates FMT, is stimulated by Ang II and affects myofibroblast motility, contractility, secretion of ECM proteins, etc. [51,52,53]. In canonical TGF-β signaling pathway, TGF-β1 binds to type I and type II serine-threonine kinase receptors (TGFβRI and TGFβRII) and initiates a cascade of molecular events [54]. The downstream molecules of TGF-β includes Smad2, Smad3, Smad4 and Smad7. Smad2 and Smad3 belong to receptor-regulated Smads (R-Smads) which are directly activated via phosphorylation by TGFβRI, forming a complex with the common mediator Smad4. This complex then translocates into the nucleus and modulates target gene transcription [55,56], whereas Smad7 functions as an inhibitory Smad (I-Smad) which antagonizes the activity of Smad2 and Smad3 [57]. In addition to canonical TGF-β signaling pathway, MAPK, Wnt/β-catenin, EGFR and Endoglins signal pathways are regulated by TGF-β/Smad signaling, resulting in a complex set of interactions known as pathway crosstalk [58,59]. Collectively, TGF-β signaling pathway is the master of cardiac fibroblast differentiation and cardiac fibrosis (Figure 3). Targeting TGF-β pathways could be a novel therapeutic strategy to manage cardiac disorders.

### 3.3. Progress on Proteomic Studies of Patients with Cardiovascular Disease

Proteins are the driving force of the cellular machinery and they control virtually all physiologic events. Proteomics is the analysis of the entire protein complement of an organism, tissue, cell or biofluid under a specific, defined set of conditions [60,61]. With the rapid advances in mass spectrometry instrumentation and breakthrough achievements in bioinformatics tool, most proteomic discoveries and efforts to date have been used to investigate the condition and location of protein expression [62,63], the ways in which proteins are modified (for example, post-translational modifications (PTMs) such as phosphorylation) [64,65], and the ways in which proteins interact with one another [66], presenting an unmatched data completeness and sensitivity.

Quantitative proteomics is a powerful approach used for both discovery and targeted proteomic analyses to understand global protein expression and modifications underlying the molecular mechanisms of biological processes and disease states. A qualitative proteomics workflow consists of cellular lysis, protein separation and digestion followed by LC-MS analysis [67,68]. Conventional proteomic studies perform qualitative or quantitative protein analysis based on three dimensions, defined by retention time, mass-to-charge ratio (m/z), and ion intensity. With the development of Trapped Ion Mobility Spectrometry (TIMS) and Parallel Accumulation–Serial Fragmentation (PASEF) techniques, molecule-specific collisional cross section (CCS) values have been introduced to increase peak capacity and confidence in compound characterization, making 4D proteomics technology the latest quantitative approach. Concretely, the TIMS device enables a unique increase in sensitivity and speed along with the additional dimension of separation—the CCS value via the scan mode termed PASEF, to accumulate and concentrate ions of a given mass and mobility [69]. In a 120 min single-shot experiment on 200 ng HeLa digest, diaPASEF identified more than 6000 proteins without matching to a library and with high quantitative reproducibility. Online PASEF further achieved a remarkable sensitivity with more than 2500 proteins identified in a 30 min run of only 10 ng HeLa digest [70]. Collectively, quantitative proteomic researches help scientists dig deeper into the complex cellular machinery with the potential to discover low-level, biologically significant proteins, or validate them in translational medical research.

Fibrosis is an extraordinarily heterogeneous process, as several stages of cardiac fibrosis exist, each with different fibrosis subtypes and a diverse composition of various cells and proteins, resulting in an exceedingly complex pathophysiology. Early insidious perturbations such as subclinical hypertension or chronic inflammation may trigger initial fibrotic events, while more dramatic triggers such as MI and myocarditis lead to scar formation and ongoing fibrosis in diseased hearts [71]. Proteomic studies using clinical specimens from patients with myocardial fibrosis aim to discover novel circulating biomarkers and decipher cellular mechanisms underlying fibrosis-associated diseases.

Heart failure is still a frequent complication of MI, thus management of MI-induced replacement fibrosis and identification of candidate proteins associated with post-MI heart failure may contribute to clinical intervention. In a New Zealand cohort, aptamer-based affinity capture plasma proteomics were utilized to measure 1305 plasma proteins at 1 month post-MI, and 212 differentially expressed plasma proteins were significantly associated with subsequent heart failure events. By correlating 96 plasma proteins with left ventricular ejection fraction measured at 4 months post-MI, researchers illustrated six top proteins potentially coregulated in post-MI heart failure: N-terminal B-type natriuretic peptide (NT-BNP) and troponin T (TNNT2), and newly emergent biomarkers, angiopoietin-2 (ANGPT2), thrombospondin-2 (THBS2), latent transforming growth factor-β binding protein-4 (LTBP4), and follistatin-related protein-3 (FSTL3), as well [72].

Many CVD risk factors such as plasma lipids, blood pressure, glucose/insulin resistance, and inflammation are aggravated by high level of fat mass [73]. Kresoja et al. have identified circulating serum biomarkers in obese patients suffering from heart failure with preserved ejection fraction (HFpEF). After adjusting for covariates, five proteins (ADM, THBS-2, Gal-9, CD4 and TRAIL-R2) were significantly elevated in the results of comparison of obese HFpEF patients to obese non-HFpEF or lean HFpEF patients [74]. Among these proteins, THBS-2 is a critical modulator of ECM homeostasis in fibrotic progression. In aging heart, absence of THBS-2 resulted in impaired Src/Akt-dependent cardiomyocyte survival [75].

## 4. Proteomic Insights into Cardiac Fibrosis

### 4.1. Understanding Cardiac Fibrosis in Animal Models

Fibrosis is frequently explored in experimental heart failure from preclinical animal models. The triggers, dynamics, and characteristics of the fibrotic process are very different among various etiologies of heart failure. Below, we characterize cardiac fibrosis in the setting of MI and pressure overload in vivo.

Replacement fibrosis is typically the fundamental process observed in MI, and surgical ligation of the coronary arteries in mice is the most common animal model of replacement fibrosis [76]. Gao et al. reported a novel rapid procedure of MI in mice inducing myocardial ischemic injury with less inflammation and higher post-MI survival rate compared with the classic method, which represented a more efficient model to facilitate studies of myocardial fibrosis [77]. Li et al. established a MI mouse model by ligation of the proximal left anterior descending coronary artery, collected heart samples at different time points after MI, and investigated the molecular changes via RNA-seq and data-independent acquisition mass spectrometry (DIA-MS) approaches [78]. Integrative analysis of proteome and transcriptome revealed that fibroblasts were the predominant participants in replacement fibrosis; moreover, proteins that functioned in cell proliferation, fibrinolysis, secretion, and immunity were significantly changed after MI. Three secreted proteins (Serpina3n, Nppa, and Anxa1) had remarkably changed at both omics levels and presented valuable potential for MI diagnosis. In detail, Serpina3n, a serine protease inhibitor, is released into the circulation during muscle atrophy [79]. *Nppa*, encoding atrial natriuretic factor (ANF), is highly expressed in the atria and ventricles during heart development. After birth, ventricular expression of Nppa is strongly downregulated [80]. During hypertrophy and heart failure, Nppa is reactivated in ventricular cardiomyocytes and serves as a highly conserved marker of heart disease [81,82]. Anxa1, a calcium-dependent phospholipid-binding protein, acts in immune system modulation and cell membrane organization [83].

TAC is another frequently used surgical model of heart failure which triggers interstitial fibrosis and perivascular fibrosis. In the TAC model, the aorta between the first (brachiocephalic artery) and second (left common carotid artery) branch was tied with a cannula, the aortic diameter is reduced to about 0.4 mm after removing the cannula, which mimics increased left ventricle pressure overload and induces cardiac fibrosis and heart failure [84,85]. The fibrotic process is closely related to mitochondrial dysfunction since heart is a highly oxygen-consuming organ. During cardiac fibrosis, the delicate balance between ROS biogenesis and ROS scavenging is disturbed by severe redox stress events, which lead to mitochondria morphological changes, mitochondrial membrane potential and structural damage, mitophagy and mitochondrial transfer disorder [86]. Research group of Peter Rabinovitch applied label-free proteomic approach to elucidate the global proteomic alterations and signal pathway changes in TAC-induced heart failure. The top pathways were in the categories of actin cytoskeleton, mitochondrial function, intermediate metabolism, glycolysis/gluconeogenesis, and citrate cycle, in parallel with the protective effects of mitochondrial-targeted peptides on the congestive heart failure phenotypes and mitochondrial damage induced by TAC [87].

To preserve the function of left ventricular, pressure overload triggers centripetal hypertrophy of the ventricular wall in the initial stage [88,89]. However, prolonged pressure overload usually leads to dilatation of the ventricular wall and interstitial fibrosis. This dynamic evolutionary process is characterized by considerable disturbance of protein turnover. Liu et al. performed label-free quantitative proteomics to illustrate the alterations of cardiac protein expression in TAC model at different time points. Compared with sham-operated mice, 101 proteins were changed significantly at 2, 4 and 12 weeks after TAC surgery. Furthermore, disturbed cardiac energy metabolism and impairment of matrix reorganization were observed during progression of cardiac hypertrophy to heart failure [90]. Collectively, animal models of cardiac fibrosis attribute enormously to characterize diagnostic biomarkers, develop novel therapeutic strategies, and explore mechanisms of fibrosis in vivo.

### 4.2. Region and Cell-Type Resolved Quantitative Proteomic Analysis of Cardiac Fibrosis

**Figure 4:**
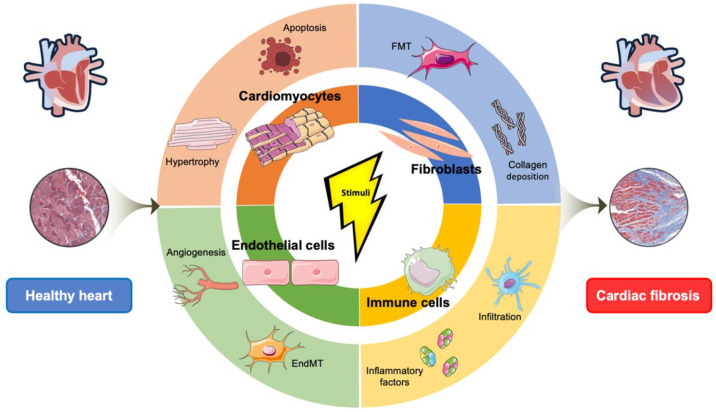
Illustration of cell-type resolved quantitative proteomic analysis of cardiac fibrosis.

Human heart is composed of diverse cell types including cardiomyocytes, cardiac fibroblasts, endothelial cells, immune cells, etc. In pathological myocardial fibrosis, these cells can be divided into two main categories: producing matrix proteins (fibroblast and myofibroblast) and secreting profibrotic mediators (macrophages, mast cells, lymphocytes, cardiomyocytes, and vascular cells) [2]. Prior to the onset of cardiac fibrosis, expression levels of various profibrotic factors are upregulated in cardiac fibroblast, and afterward shift their phenotype to myofibroblasts with increasing proliferation and collagen secretion capacity during pressure overload [11]. Previous studies have focused on elucidating potential causal roles of individual genetic variants in CVD progression [91,92] and defining global changes in patients with CVDs between human myocardial tissues [93,94] or plasma samples [72,95] via mass spectrometry-centered approaches. It is desirable to gain deeper insights into the molecular characteristics of cardiac fibrosis at the cell type- and heart region-resolved level. In particular, characterization of the healthy state of human heart is an important starting point, and quantification of anatomically resolved or cell-type specific variations would provide valuable information about disease pathogenesis and potentially uncover pathways for therapeutic intervention for cardiac fibrosis (Figure 4).

Proteomics studies have largely utilized homogenized human cardiac tissue; however, proteomic profiling with spatial resolution or isolation of one or multiple cell types from a heterogeneous population has the potential to define the complex cellular and molecular architecture of the human heart in health and disease [96,97]. The most widely used cell isolation and separation techniques can be broadly classified as based on adherence, morphology (density/size) and antibody binding. The Langendorff retrograde perfusion is the most efficient method for isolation of adult cardiomyocytes [98,99]. However, the drawbacks of perfusion, including the complex of Langendorff system preparation and the time-consuming perfused digestion of heart with collagenase solution, could hydrolyze protein molecules on the cell surface and limit the large-scale clinical applications of this isolation protocol. Recently, the acquisition of primary cardiac fibroblasts and microvascular endothelial cells is usually performed by collagenase–trypsin digestion method, then purified by differential adhesion or magnetic bead sorting [100,101].

#### 4.2.1. Cardiomyocytes

Inflammatory response induced by the damaged cardiomyocytes is the main initiating factor for the fibrotic response. Given the poor cardiac regenerative capacity of adult mammals, considerable efforts have been made in attempts to somehow increase the tolerance of cardiomyocytes to injurious conditions. Wojtkiewicz et al. described the first method for cardiomyocyte isolation from cryopreserved human cardiac tissue followed by flow cytometry for purity assessment. Bottom-up proteomic analysis of isolated cardiomyocytes provided a deeper view than tissue homogenate, and quantitative comparisons revealed differences among heart chambers [102]. In a newly published article, Lu and colleagues utilized proximity proteomics in living hearts to identify proteins that were abundant around dyads, an architecture which is essential for cardiac excitation–contraction coupling. Cardiomyopathy-associated protein 5 (CMYA5), a protein selectively expressed in cardiac and skeletal muscle which co-locates with dyad architecture, was validated as a dyadic protein. Ablation of CMYA5 affected dyad architecture, dyad location at Z-lines, and junctional sarcoplasmic reticulum Ca^2+^ release, resulting in cardiac dysfunction and inability to tolerate pressure overload [103].

Diverse cells in in cardiac tissues sense mechanical stimuli and physical environment, then integrate functional responses to these external stimuli. Different elements of the cells must be coordinated to maintain structural integrity and ensure cellular sensing of external forces and mechanical stimuli. These stimuli are subsequently transmitted to the nucleus, leading to profound alterations in chromatin structure and accessibility [104,105]. The cell–cell crosstalk directs integrated biological responses to various stimuli, and secreted proteins are critical for communications between neighboring cells in living heart. Secretomics, which is a subset of proteomics, has emerged as a recognized field in recent years. In a novel study, investigators combined stable isotope tagging and click chemistry with subsequent mass spectrometry analysis to develop a distinctive secretome analysis of primary cardiomyocytes. A total of 1026 proteins were identified to be secreted by cardiomyocytes suffered from serum-free starvation. After the analysis of hypoxia-induced cardiomyocytes secretion, PCSK6 was shown to be involved in cardiac remodeling following an acute MI [106].

#### 4.2.2. Cardiac Fibroblasts

Shah et al. conducted cell-resolved proteomic analysis to identify differentially altered proteins and signaling pathways between cardiac fibroblasts isolated from sham and infarcted murine hearts. Interestingly, 15 differentially expressed proteins were identified between cardiac fibroblasts from the remote and infarct regions of injured hearts, with the magnitude of change reflecting their relative proximity to the site of injury [107].

Zhang et al. carried out large-scale quantitative proteomics using infarcted mouse hearts, fibrotic livers generated by CCl4, bleomycin-treated lungs, as well as sham and vehicle-treated control tissues. Each sample contained approximately 4600 quantifiable proteins and about 800 proteins were differently expressed in the infarcted heart compared to the sham-heart. Among these differential proteins, cartilage intermediate layer protein 1 (Cilp1) was expressed predominantly in cardiac fibroblasts and upregulated in animal models of adverse cardiac remodeling. Compared with wild-type mice, Cilp1 knock out mice had better cardiac function, reduced number of immune cells and myofibroblasts, attenuated collagen expression, and enhanced microvascular survival after MI injury, which indicated Cilp1 could be a therapeutic target for cardiac fibrosis [108].

Kalyanasundaram et al. explored the function of adult human sinoatrial node (SAN)-specific fibrosis in atrial fibrosis and arrhythmias. Cardiac fibroblasts from two anatomical regions, including central intramural SAN pacemaker compartment and right atria (RA), were first isolated from nonfailing (non-HF) and HF human hearts. Fibroblasts were then cultivated and treated with or without TGFβ1. Proteomic signatures and signaling pathways associated with ECM flexibility, stiffness, focal adhesion, and metabolism were uniquely affected by HF or TGFβ1 treatment in SAN and RA fibroblasts. SAN fibroblasts presented robust myofibroblast differentiation and fibronectin secretion in HF human hearts, and POSTN-positive fibrotic islands increased in human HF SAN, which led to intranodal structural barriers interrupting normal automaticity and conduction, whereas molecular and protein markers were distinguished from RA fibroblasts in non-HF SAN fibroblasts, indicating these proteomic datasets could be used to identify novel SAN fibroblast-specific targets to develop antifibrotic strategies [109].

Although activated fibroblasts have been the primary effector cells in the fibrotic heart by producing ECM proteins directly, immune cells, vascular cells and cardiomyocytes are also involved in the process of cardiac fibrosis via different pathways. Research group of Joerg Heineke revealed a novel role for the cardiogenic transcription factors GATA-4 and GATA-6 in cardiac fibroblasts, where both proteins promoted myocardial adaptation to pressure overload though enhancing cardiac angiogenesis. Specifically, mice with double deletion of Gata4 and Gata6 in activated cardiac fibroblasts reduced the capillary density in the heart after pressure overload. By co-culture system with endothelial cells and Gata4/6-deprived cardiac fibroblasts, endothelial cells exerted reduced migration and tube formation, and loss of Gata4/6 in vitro reduced angiogenic response. This finding was supported by proteomic profiling of the extracellular matrix from whole hearts of Gata4/6fl and Gata4/6fl-Per-Cre mice, which revealed a significantly higher level of the CD36, an anti-angiogenic glycoprotein, in mice with fibroblast-specific downregulation of Gata4 and Gata6 [110].

#### 4.2.3. Endothelial Cells

Emerging evidence indicates that endothelial cells perform a fundamental role in the pathologic remodeling of cardiac fibrosis. Myocardial interstitial has a more intensive vascular and capillary network to maintain the high energy consumption and metabolic demands of the mammalian heart, which is the major manifestation of endothelial cells (ECs). During cardiac fibrosis, a number of growth factors act on ECs, driving them to obtain certain characteristics of mesenchymal cells and to lose those of ECs, including loss of tight junctions, increased motility, and elevated secretion of ECM proteins. This dynamic process is known as the endothelial-to-mesenchymal transition (EndMT) [111]. Notably, changes in the microenvironment of fibrotic organ trigger modified gene expression in ECs. Cells then lose cell–cell junctions, degrade the basement membrane, and migrate out into the perivascular surroundings, which display fibroblast-like morphology and functions [112,113].

ECs can contribute to fibrosis by acting as an important source of myofibroblasts via EndoMT, which account for approximately 30% in a cardiac fibrosis model induced by ascending aortic constriction [45]. Additionally, by communicating with cardiomyocytes, fibroblasts and inflammatory cells, cardiac microvascular endothelial cells (MiVEC) secrete multiple profibrotic mediators and pro-inflammatory cytokines to promote pathophysiological processes in cardiac fibrosis and matrix remodeling. Research group of Stefan Janssens characterized transcriptional and metabolic adaptation of cardiac MiVEC to pressure overload-induced heart disease in mice and patients with severe aortic stenosis. Time course analysis of mouse cardiac MiVEC revealed early and sustained upregulation of collagens and *Cilp*, whereas other TGFβ-modulating genes, *Adamtsl2* and *Thbs4*, were upregulated only at a later stage of TAC model. Pressure overload induces a major fibrotic rewiring of MiVEC from essential energy suppliers and effectors of angiogenesis to a matrix secretory phenotype, resulting in excess interstitial fibrosis and impaired angiogenesis, predominantly in female patients who suffered chronic left ventricle pressure overload [114].

Endothelial nitric oxide synthase (eNOS), an essential mediator for maintaining vascular function, is tightly regulated at protein levels which requires protein–protein interactions. Stable isotope labeling by amino acids in cell culture (SILAC)-based quantitative proteomic approach was applied to identify several eNOS interactors, including the protein plasminogen activator inhibitor-1 (PAI-1). Subsequently, PAI-1 was uncovered as a potent negative regulator of eNOS function; disruption of eNOS–PAI-1 binding resulted in increasing NO generation and vasodilation in vivo [115].

#### 4.2.4. Extracellular Matrix Remodeling

The extracellular matrix (ECM) is a major component of the cellular microenvironment. In addition to cellular-level alterations, ECM remodeling is another critical characteristic of cardiac fibrosis, where ECM components are deposited, degraded, or otherwise modified. Cardiac ECM, a significant medium in the mechanical connection within the myocardium, is a highly dynamic structure composed of many elements including collagens, fibronectin, glycoproteins, and metalloproteinases (MMPs)/tissue inhibitors of metalloproteinases (TIMPs). Excessive deposition of ECM disrupts cardiac architecture, myocardial excitation–contraction coupling, systolic and diastolic function, all of which lead to adverse cardiovascular outcomes [116,117]. Understanding quantitative and qualitative changes in the ECM may contribute to deciphering of new mechanisms of cardiac fibrosis with diagnostic and/or therapeutic potential.

Barallobre-Barreiro et al. used 3-step ECM extraction procedure to extract left ventricle ECM proteins from patients with ischemic heart failure displayed focal replacement fibrosis (n = 5), patients with heart failure of nonischemic origin showing left ventricular (LV) dilatation (n = 10) and donor hearts (n = 6). Label-free tandem mass spectrometry analyses were performed to find that the proteoglycans ASPN (asporin), BGN (biglycan), DCN (decorin), LUM (lumican), OGN (mimecan, osteoglycin), PRELP (prolargin), and VCAN (versican) were significantly deposited in patient’s hearts with ischemic HF [118]. In 2012, this group reported ECM proteome remodeling in a porcine model of ischemia/reperfusion injury. By using LC-MS/MS approach, several ECM proteins, including cartilage intermediate layer protein 1 (CILP1), matrilin-4 (MATN4), extracellular adipocyte enhancer binding protein 1 (AEBP1), collagen α-1(XIV), and several members of the small leucine-rich proteoglycan family (e.g., asporin and prolargin), were first shown to contribute to cardiac remodeling. Bioinformatic analysis based on differential ECM proteins further delineated features of different stages of cardiac remodeling [119].

## 5. Conclusions

Cardiac fibrosis poses a grievous threat to human health, thus more attention should be directed to explore the pathophysiological mechanism of cardiac fibrosis and search for circulating biomarkers and therapeutic strategies. This review emphasizes the current and future roles of mass spectrometry-based quantitative proteomics in deciphering molecular mechanisms underlying cardiac fibrosis; quantitative accuracy to date strongly benefits from the 4th mobility dimension. Most importantly, spatial and cell-type resolved quantitative proteomic analysis using clinical specimens and animal models can provide the excellent capability to track pathological changes and dig deeper into the complex cellular machinery with the potential to discover low-level, biologically significant proteins, or validate them in translational medical research.

## Data Availability

Not applicable.
